# MicroRNA-653-5p Promotes Gastric Cancer Proliferation and Metastasis by Targeting the SOCS6-STAT3 Pathway

**DOI:** 10.3389/fmolb.2021.655580

**Published:** 2021-04-15

**Authors:** Zengliang Li, Hao Fan, Wangwang Chen, Jian Xiao, Xiang Ma, Peidong Ni, Zekuan Xu, Li Yang

**Affiliations:** ^1^Department of General Surgery, The First Affiliated Hospital of Nanjing Medical University, Nanjing, China; ^2^Department of General Surgery, Liyang People’s Hospital, Liyang Branch Hospital of Jiangsu Province Hospital, Liyang, China

**Keywords:** gastric cancer, miR-653-5p, SOCS6, JAK2/STAT3 pathway, cancer prognosis

## Abstract

MicroRNAs (miRNAs) are emerging as significant regulators of the tumorigenesis of gastric cancer (GC), and may be effective biomarkers for diagnosis, prognosis, and therapeutic targeting for GC. In this study, miR-653-5p was found to be significantly upregulated in GC tissues, serum, and cell lines and was strongly associated with poor prognosis in patients with GC. Furthermore, miR-653-5p promoted GC cell proliferation and metastasis *in vivo* and *in vitro*. Suppressor of cytokine signaling 6 (SOCS6) was directly targeted by miR-653-5p, and SOCS6 attenuated miR-653-5p-mediated GC cell growth, migration, and invasion. In addition, SOCS6-mediated inactivation of the Janus kinase 2/signal transducer and activator of transcription 3 (JAK2/STAT3) signaling pathway was also reversed by the administration of miR-653-5p. The findings from this study support a novel regulatory axis between miR-653-5p, SOCS6, and JAK2/STAT3 that may be a target for diagnosis and therapeutic intervention for GC.

## Introduction

Gastric cancer (GC) is one of the most prevalent and deadly cancer types worldwide, particularly in East Asia (Sung et al., 2021). The prognosis for patients with GC is poor due to diagnosis at advanced stages, which highlights the necessity for the identification of effective diagnostic and prognostic biomarkers (Digklia and Wagner, 2016). MicroRNAs (miRNAs), first identified in *Caenorhabditis elegans* in 1993 (Lee et al., 1993), are small (20–22 nucleotides in length) endogenous non-coding RNAs that are ultimately assembled into RNA-induced silencing complex (RISC) with the AGO2 protein. miRNAs then bind complementary mRNA sequences to repress expression *via* degradation of posttranscriptional mRNA or inhibition of translation (Ha and Kim, 2014). Dysregulated expression of miRNAs has been linked to various diseases, including onset and progression of different types of cancer (Lee and Dutta, 2009).

The recent advent of genomics and novel sequencing approaches has resulted in the identification of key miRNAs involved in proliferation, metastasis, and drug resistance of GC cells through actions as oncogenes or tumor suppressors (Wu et al., 2010; Liu et al., 2020). For example, a study showed that loss of miR-29c expression, which acts as a tumor suppressor *via* direct targeting ITGB1, was an early event in the initiation of gastric carcinogenesis (Han et al., 2015). Intriguingly, stable miRNAs have been detected in bodily fluids, particularly in blood, plasma, serum, and saliva, which further strengthens the potential of miRNAs as non-invasive biomarkers (Huang et al., 2017). Moreover, the therapeutic potential of miRNAs in cancer has received increased attention (Rupaimoole and Slack, 2017). Therefore, systematic investigation of miRNA signatures in GC and characterization of the underlying mechanisms of miRNAs may aid in the development of targeted treatment strategies with improved clinical efficacy for individual patients with GC.

In this study, miR-653-5p was selected through the analysis of online databases as a candidate for the promotion of GC cell proliferation and metastasis *in vivo* and *in vitro*. In addition, analysis of miRNA–mRNA interactions indicated that suppressor of cytokine signaling 6 (SOCS6), a member of the SOCS family of proteins that act as negative regulators of cytokine and growth factor signaling, was the downstream target of miR-653-5p. The SOCS family of proteins is comprised of eight members (CIS, SOCS1–7) that contain a C-terminal SOCS box and an SH2 domain preceded by an N-terminal region of variable length (Krebs and Hilton, 2001). Studies have focused primarily on SOCS1 and SOCS3 and have shown that these proteins inhibit the JAK-STAT pathways, which are activated by cytokines and other molecules (Croker et al., 2008). However, the role of SOCS6 in the regulation of JAK-STAT signaling is unclear and requires further characterization (Nicholson et al., 1999; Shen et al., 2015). However, studies have shown that SOCS6 is involved in insulin resistance (Mooney et al., 2001), KIT receptor signaling (Bayle et al., 2004), carcinogenesis (Lin et al., 2013), and is a component of the CRL5SOCS6 ubiquitin ligase (Teckchandani and Cooper, 2016). Our study showed that miR-653-5p-mediated GC progression and metastasis through modulation of the SOCS6-STAT3 signaling pathway, highlighting potential novel diagnostic and therapeutic targets for GC.

## Materials and Methods

### Tissue Samples

Gastric cancer tissues and corresponding adjacent non-tumor tissues were collected from 80 patients with GC who underwent radical gastrectomy at the First Affiliated Hospital of Nanjing Medical University. All patients were newly diagnosed with GC and had no histories of other cancers. All of the extracted tissues were examined by experienced pathologists, and GC diagnosis was confirmed by the consensus of at least two pathologists. The collected tissue samples were immediately snap-frozen in liquid nitrogen and stored at −80°C until analysis. Clinicopathological variables, such as tumor site, depth of invasion, lymph node metastasis, and TNM stages, were obtained from patient medical records. This study was approved by the Ethics Committee of the First Affiliated Hospital of Nanjing Medical University, and informed consent was obtained from each subject.

### Cell Lines and Cell Culture

Five human GC cell lines and a human normal gastric epithelial cell line (GES-1) were obtained from the Cell Bank of the Chinese Academy of Sciences (Shanghai, China). The GES-1, MGC803, BGC823, SGC7901, and MKN45 cell lines were maintained in the RPMI 1640 medium, and the AGS cell line was cultured in the F12K medium. All media were supplemented with 10% fetal bovine serum and 1% ampicillin–streptomycin (Wisent, Montreal, QC, Canada). The cells were incubated at 37°C in a humidified atmosphere containing 5% CO_2_.

### RNA Extraction and Quantitative Real-Time PCR (qRT-PCR)

Total RNA was isolated from cultured cells or frozen tissues using TRIzol reagent (Invitrogen, Carlsbad, CA, United States) and then quantified using a NanoDrop 2000 Spectrophotometer to measure the absorbance at 260 nm (Thermo Fisher Scientific, United States). The extracted mRNA was reverse-transcribed using a Revert Aid First Strand cDNA Synthesis Kit (Thermo Fisher Scientific, MA, United States) for miRNA or using a PrimeScript^TM^ RT reagent Kit (Takara, Kyoto, Japan) for other RNA species, according to the instructions of the manufacturer. Quantitative PCR was performed using SYBR Green PCR Master Mix (Vazyme, Nanjing, China) by following a standard quantitative PCR procedure. Gene expression was normalized to GAPDH or U6. The primers used in this study are summarized in Supplementary Table 1.

### Cell Transfection and Vector Construction

First, the LV2-hsa-miR-653-5p-mimic vector (miR-653-5p-mimic) and the LV2-hsa-miR-653-5p-inhibitor vector (miR-653-5p-inhibitor) (GenePharma, Shanghai, China) were constructed; then, transduced into GC cells according to the instructions of the manufacturer. The LV2 empty lentiviral construct was used as a negative control. Biologically active short hairpin RNAs (shRNA) targeting SOCS6 and a lentiviral vector containing SOCS6 sequences were subcloned and amplified using a lentiviral expression vector (GenePharma, Shanghai, China). Stable cell lines were obtained by treatment with 2 μg/ml puromycin (Sigma-Aldrich, St. Louis, MO, United States) for about 3 weeks.

### Cell Proliferation Assays

Cell Counting Kit-8 (CCK-8) assay, colony formation assay, and EdU incorporation assay were used to evaluate the effects on GC cell proliferation. These experiments were performed as previously described (Lin et al., 2019).

### Cell Migration and Invasion Assays

Wound healing and transwell assays were used to determine the effects of treatment conditions on GC migration and invasion. Linear scratch wounds were created using a 200 μL sterile pipette tip. The cells were cultured in a serum-free medium, and the wounds were imaged at the same position at multiple time points. Transwell assays were conducted using a 6.5 mm chamber with 8 μm pores (Millipore, Darmstadt, Germany) in the presence or absence of Matrigel coating (50 μg/ml, BD Biosciences, United States). This procedure was performed as previously described (Lin et al., 2019).

### Immunofluorescence Analysis

Transfected cells were washed with cold PBS, fixed with 4% PFA for 15 min, washed three times with PBS, and then permeabilized with 0.5% Triton X-100 (PBS) at room temperature for 10 min. Non-specific binding was blocked using normal goat serum (Invitrogen). Cell lysates were incubated overnight at 4°C with primary antibodies against vimentin (1:100, Cell Signaling Technology, United States) and E-cadherin (1:100, Proteintech, Wuhan, China) and stained with fluorescent-conjugated secondary antibodies (1:100, Beyotime, Shanghai, China). Nuclei were stained with DAPI for 5 min (Beyotime, Shanghai, China). Images were captured at appropriate magnification using a fluorescence microscope (Ti2-E, Nikon, Japan). Protein expression was quantified based on staining intensity using ImageJ software.

### Western Blot Analysis

Total protein from GC cells and primary tissues was extracted using the RIPA lysis buffer (Beyotime, Shanghai, China). Extracted proteins were separated by sodium dodecyl (SDS)-PAGE and transferred to a PVDF membrane. The membranes were blocked and incubated with specific primary antibodies and secondary antibodies. The relative expression levels of the proteins were determined using an ECL detection system. The primary antibodies used are listed in Supplementary Table 2.

### Animal Experiments

Stably transfected BGC823 and SGC7901 cells (1 × 10^6^ cells/100 μl PBS) were subcutaneously injected into the flanks of 4-week-old female BALB/c nude mice. Tumor volumes were measured every week for about 5 weeks, after which the mice were euthanized. For the metastatic model, transfected GC cells (0.5 × 10^6^ cells) were suspended in 50 μl of PBS and then injected into the tail veins of randomly assigned individual mice. At 4 weeks postimplantation, the mice were euthanized, and the lungs were stained with H&E to allow for clone number counting. Prior to being euthanized, D-luciferin (Caliper Life Sciences, Waltham, MA, United States) was used to evaluate the presence of distant lung metastases using an *In Vivo* Imaging System (IVIS) (Caliper life Sciences, Hopkinton, MA, United States).

### Dual-Luciferase Reporter Assay

A fragment of the wild type (WT) SOCS6 3′-UTR and a mutated (mt) SOCS6 3′-UTR were cloned into the downstream region of the luciferase gene in the pGL3-basic luciferase vector (Promega, Madison, WI, United States). The luciferase reporter vectors were transfected into BGC823 cells with a miRNA mimic or a control. Luciferase activity was measured using a dual-luciferase reporter assay system (Promega, Madison, WI, United States). Firefly luciferase activity was normalized against Renilla luciferase activity.

### Immunohistochemistry (IHC) Analysis of Human GC Tissues

Immunohistochemical staining of patient tissue sections was performed as previously described (Jiang et al., 2019). The expression of SOCS6 in normal and malignant specimens was evaluated.

### Bioinformatic Analysis

To select functional miRNAs, OncomiR^1^, The Cancer Genome Atlas Program (TCGA) database^2^, and GSE106817^3^ were used. The GSE106817 dataset contains serum miRNA profiles from 4,046 women, including 115 GC serum samples, 2,759 non-cancerous control samples, and 1,172 samples of other cancer types. The associations of miR-653-5p and SOCS6 with GC prognosis were determined using KM plotter^4^. Four online tools, namely TargetScan^5^, Starbase^6^, miRWalk^7^, and TargetMiner^8^, were used to determine the miRNA target genes.

### Statistics Analysis

For statistical comparisons, one-way analysis of variance and the Wilcoxon test were performed for comparisons among multiple groups. Comparisons between the two groups were analyzed using two-tailed Student’s *t*-tests. The correlation between miR-653-5p expression and clinicopathological parameters was assessed using the chi-squared test. The Pearson correlation coefficient was used to evaluate the correlation between miR-612 and SOCS6. The data are expressed as the mean ± SD unless otherwise specified, and *p* < 0.05 was considered statistically significant. All statistical analyses were performed using SPSS v22.0 and GraphPad Prism 6. Each experiment was repeated three times as appropriate.

## Results

### Upregulation of miR-653-5p Was Associated With Poor Prognosis in Patients With GC

**FIGURE 1:**
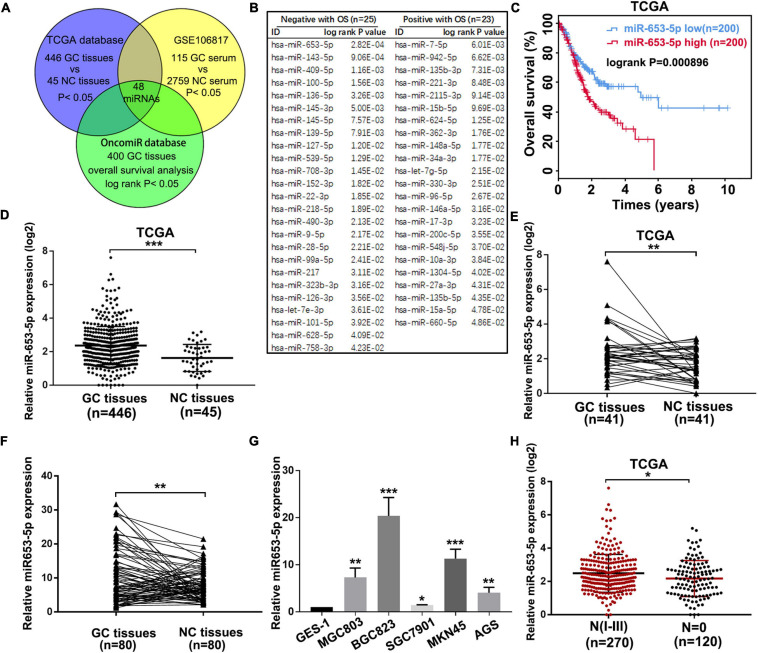
MiR-653-5p was upregulated in gastric cancer (GC) and was associated with poor prognosis. **(A)** Potential functional microRNAs (miRNAs) were predicted through analysis of online databases. **(B)** The list of prognosis-related miRNAs with high-risk scores. **(C)** Kaplan–Meier survival curve for miR-653-5p. **(D)** Relative expression of miR-653-5p in GC tissues obtained from The Cancer Genome Atlas Program (TCGA) database. **(E)** Relative expression of miR-653-5p in 41 paired GC tissues from the TCGA database. **(F)** The expression levels of miR-653-5p in 80 GC tissues compared with those in adjacent normal tissues. **(G)** Relative expression of miR-653-5p in GC cell lines. **(H)** Expression patterns of miR-653-5p based on N stages from the TCGA database. Error bars show the mean ± SD. *, *p* < 0.05; **, *p* < 0.01; ***, *p* < 0.001.

Due to the important role of miRNAs in GC tumorigenesis and the value of miRNAs in cancer diagnosis, prognosis, and therapy, we performed an integrated analysis to identify potential prognostic miRNAs, which were dysregulated in both GC tissues and serum of patients with GC. First, we identified 109 miRNAs, which were significantly associated with GC prognosis using the online tool OncomiR (Wong et al., 2018). To determine expression profiles of these miRNAs in GC tissues and serum of patients with GC, the online databases TCGA and GSE106817 were evaluated, and 48 miRNAs were selected (Figure 1A and Supplementary Figures 1A,B). As shown in Figure 1B, 25 miRNAs, including miR-653-5p, were associated with poor overall GC survival, while the remaining 23 miRNAs predicted a better prognosis for patients with GC. Based on the significantly increased expression, and a strong association with poor prognosis, of miR-653-5p in GC as determined *via* analysis of the TCGA database (Figures 1C–E and Supplementary Figure 1C), we further evaluated the expression level of miR-653-5p in 80 paired GC tissues and adjacent normal tissues using reverse transcription and qRT-PCR. The results showed that miR-653-5p was overexpressed in GC tissues (Figure 1F). Furthermore, the expression of miR-653-5p was upregulated in GC cell lines compared with that in control GES-1 cells (Figure 1G). In addition, higher expression levels of miR-653-5p were associated with lymph node metastasis, as determined by analysis of the TCGA database (Figure 1H). To evaluate associations with GC clinicopathological features, the cohort was categorized into low and high miR-653-5p groups. The results showed that high expression of miR-653-5p was associated with lymph node metastasis (*p* = 0.002) and advanced TNM stages (*p* = 0.044; Supplementary Table 3). These data indicated that miR-653-5p was upregulated in GC and was involved in GC progression.

### MiR-653-5p Promoted Gastric Cancer Proliferation and Metastasis *in vitro*

**FIGURE 2:**
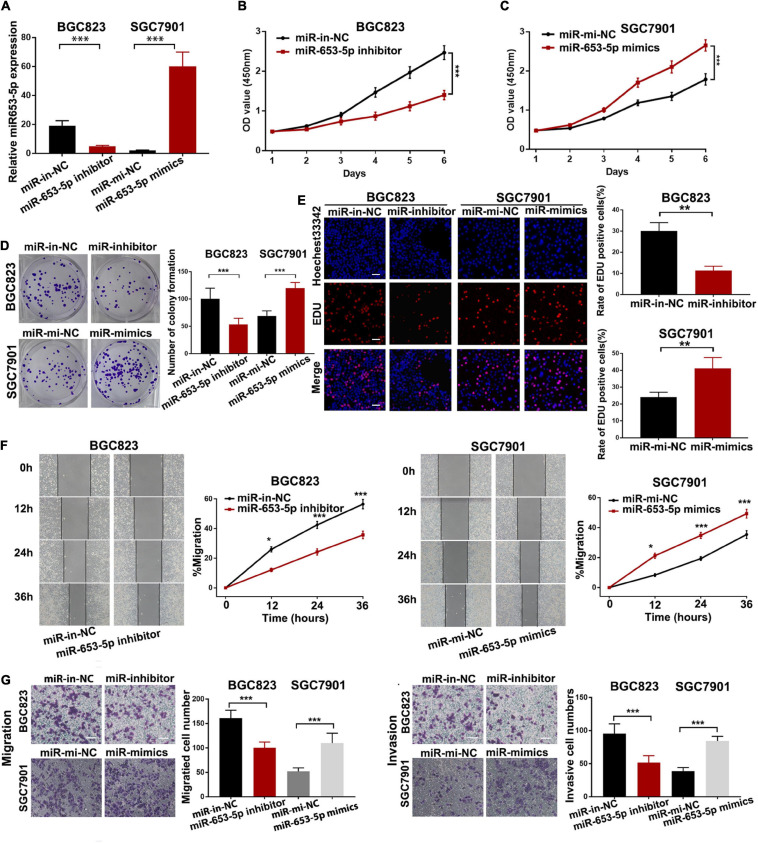
MiR-653-5p promoted gastric cancer cell proliferation, migration, and invasion *in vitro*. **(A)** MiR-653-5p was silenced or overexpressed using miRNA inhibitors or mimics. **(B)** Depletion of miR-653-5p inhibited GC cell growth. **(C)** Overexpression of miR-653-5p promoted GC cell growth in SGC7901 cells. **(D,E)** Evaluation of GC cell proliferation using colony formation assays **(D)** and EdU incorporation assays **(E)** for miR-653-5p; scale bar = 100 μm. **(F,G)** Detection of GC cell migration and invasion using the wound healing assay **(F)** and transwell assays **(G)**; scale bar = 100 μm. Error bars show the mean ± SD. *, *p* < 0.05; **, *p* < 0.01; ***, *p* < 0.001.

**FIGURE 3:**
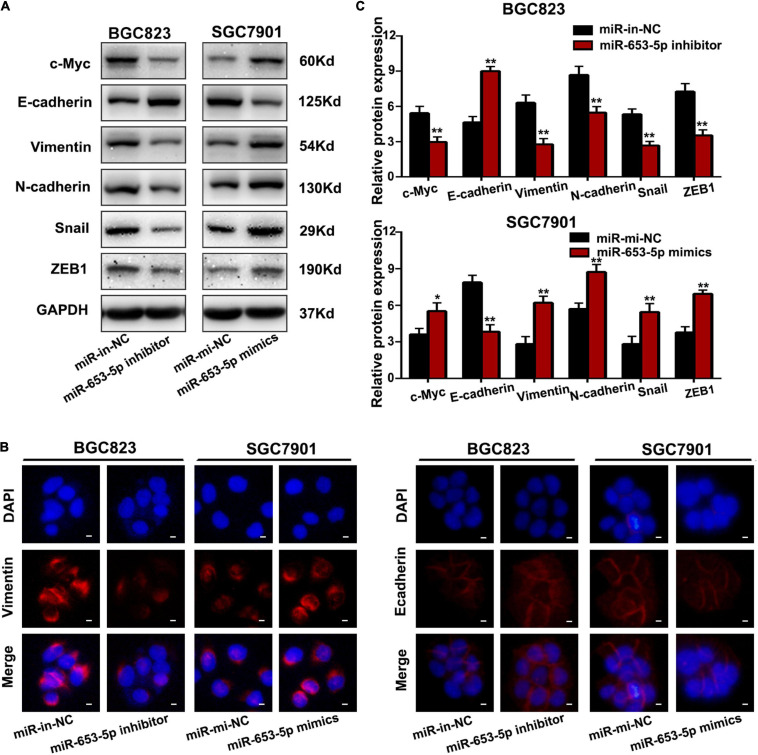
MiR-653-5p promoted the EMT process in GC cells. **(A)** Protein levels of c-Myc and epithelial to mesenchymal transition (EMT)-related proteins were measured using western blot. **(B,C)** The intensity of immunofluorescence in GC cells using primary antibodies against vimentin **(left panel)** and E-cadherin **(right panel)** was quantified; scale bar = 25 μm. Error bars show the mean ± SD. *, *p* < 0.05; **, *p* < 0.01; ***, *p* < 0.001.

To investigate the effect of miR-653-5p on GC tumorigenesis, we evaluated BGC823 and SGC7901 cell lines, which have relatively high and low miR-653-5p expression, respectively. The expression of miR-653-5p was efficiently knocked down by a miRNA inhibitor and was significantly overexpressed using a miRNA mimic (Figure 2A). Downregulation of miR-653-5p significantly inhibited growth of BGC823 cells, while overexpression of miR-653-5p-induced the proliferation of SGC7901 cells (Figures 2B,C). Similar results were obtained from colony formation assays (Figure 2D). Furthermore, EdU incorporation assays showed that EdU-positive cell numbers were significantly reduced following the depletion of miR-653-5p in BGC823 cells. In contrast, overexpression of miR-653-5p resulted in an increase in EdU-positive cells (Figure 2E). GC cells can also gain migratory abilities, which can result in metastasis. Wound healing assays were used to evaluate the effect of miR-653-5p on GC cell migration. The results showed that miR-653-5p promoted GC cell migration (Figure 2F). In addition, transwell assays with or without Matrigel coating showed that knockdown of miR-653-5p resulted in less migration than that observed in control cells. In contrast, overexpression of miR-653-5p was associated with an increase in migratory cell numbers (Figure 2G). To characterize the molecular mechanisms of these effects, the extent to which treated GC cells underwent the epithelial to mesenchymal transition (EMT) was evaluated. As shown in Figure 3A, downregulation of miR-653-5p resulted in an increased expression of the epithelial marker E-cadherin in BGC823 cells and a decreased expression of the mesenchymal markers vimentin, N-cadherin, snail, and ZEB1. Overexpression of miR-653-5p in SGC7901 cells reversed the changes observed in BGC823 cells with downregulated miR-653-5p. The expression of c-Myc was decreased in response to miR-653-5p knockdown (Figure 3A). Furthermore, immunofluorescence analysis showed that vimentin expression was decreased in BGC823 cells treated with a miR-653-5p inhibitor and was enhanced in response to the overexpression of miR-653-5p (Figure 3B). Immunofluorescence analysis showed that E-cadherin expression was increased in BGC823 cells treated with a miR653-5p inhibitor, and decreased in response to overexpression of miR-653-5p (Figure 3C). These results showed that miR-653-5p may contribute to the development and progression of GC.

### MiR-653-5p Induced Gastric Cancer Tumorigenesis and Metastasis *in vivo*

**FIGURE 4:**
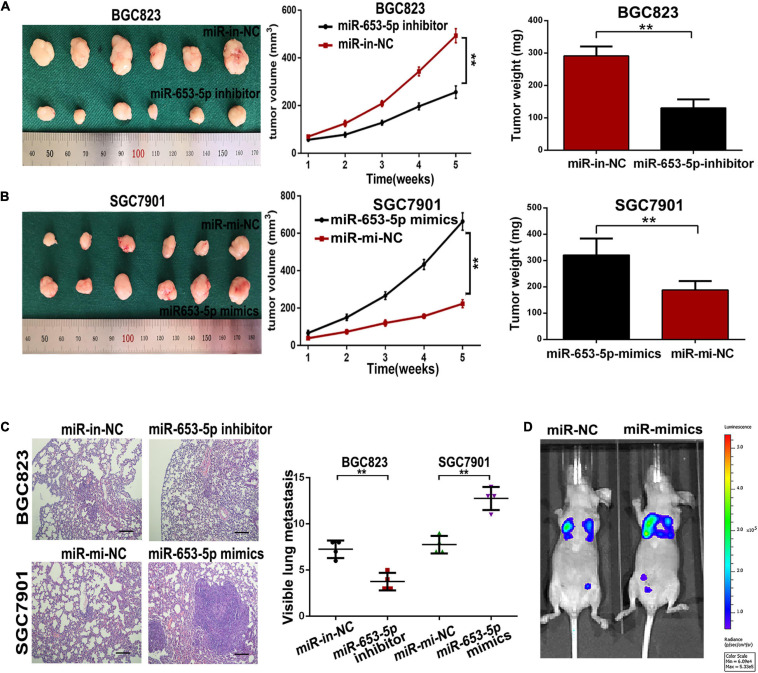
MiR-653-5p promoted gastric cancer proliferation and metastasis *in vivo*. **(A,B)** Transplanted tumor nodules were harvested **(left panel)** and weighed **(right panel)**, and growth curves were generated **(middle panel)**, following injection with BGC823 cells **(A)** or SGC7901 cells **(B)**. **(C)** Lungs with tumor nodules in each metastatic animal model are shown (200×; scale bar = 100 μm). **(D)** Representative photographs of tumors from each group were taken using an IVIS Imaging System. Error bars show the mean ± SD. *, *p* < 0.05; **, *p* < 0.01; ***, *p* < 0.001.

To evaluate the role of miR-653-5p in GC development and metastasis *in vivo*, cells were transfected with miRNA mimics or inhibitors and directly injected into the flanks of 4-week-old female nude mice. Tumor sizes and final tumor weights in implanted tumors were significantly lower in animals injected with miR-653-5p-silenced BGC823 cells (Figure 4A). In contrast, tumor sizes and weights were decreased in animals injected with cells that overexpressed miR-653-5p (Figure 4B). In parallel, we established a lung metastatic model by inoculating GC cells directly into the tail veins of nude mice. As shown in Figures 4C,D, the lung metastatic burden was decreased in the miR-653-5p-silenced group compared with that in the control group and was increased with increased expression of miR-653-5p. Moreover, the bioluminescent signals were higher in the group treated with a miR-653-5p mimic than those in the control group. In contrast, a miR-653-5p inhibitor suppressed the bioluminescent signals (Supplementary Figure 1D). These results further highlighted the functional significance of miR-653-5p in GC progression.

### SOCS6 Was Directly Targeted by miR-653-5p

**FIGURE 5:**
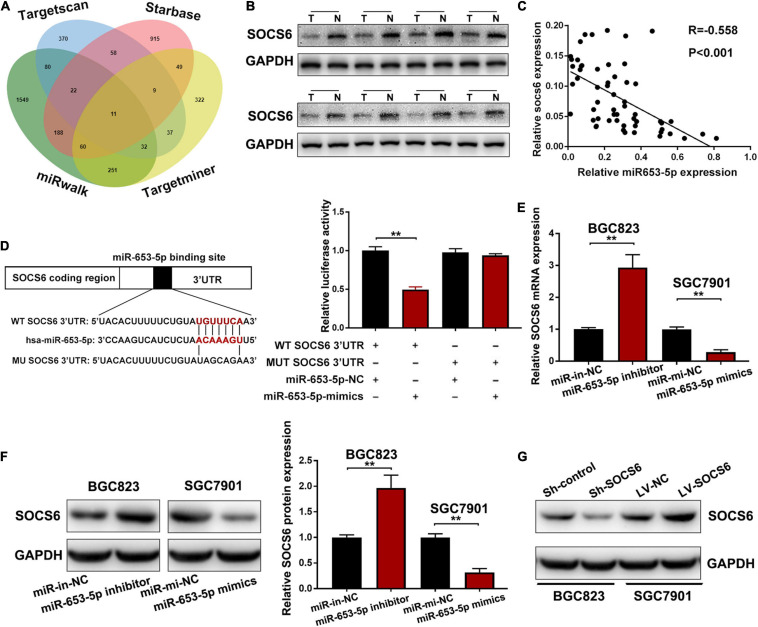
Suppressor of cytokine signaling 6 (SOCS6) was directly targeted by miR-653-5p. **(A)** Venn diagram showing the downstream targets of miR-653-5p. **(B)** Western blot analysis of SOCS6 expression in GC tissues. **(C)** Pearson correlation analysis between miR-653-5p and SOCS6 in 58 GC tissues. **(D)** Luciferase reporters containing mutated or wild-type SOCS6 sequences were co-transfected with a miR-653-5p mimic or a control into BGC823 cells as indicated. **(E,F)** Relative RNA **(E)** and protein **(F)** expression levels of SOCS6 in treated GC cells. **(G)** Western blot analysis of SOCS6 expression in GC cells transfected with corresponding vectors. Error bars show the mean ± SD. *, *p* < 0.05; **, *p* < 0.01; ***, *p* < 0.001.

Analysis of miRNA–mRNA interactions and the inverse expression patterns hidden in them is important in miRNA research. In this study, TargetScan (Agarwal et al., 2015), Starbase (Li et al., 2014), miRWalk, and TargetMiner showed that the 3′UTR of SOCS6 contained conserved putative miR-653-5p-targeting sites (Figure 5A). In addition, significant downregulation of SOCS6 was observed in GC tissues (Figure 5B), and high expression levels of SOCS6 were associated with better prognosis (HR = 0.53, 95% CI = 0.43-0.64), even in patients with advanced TNM stages (Supplementary Figure 2). Furthermore, a significant inverse correlation between miR-653-5p and SOCS6 was observed in 58 GC tissues, as determined by qRT-PCR (*R* = −0.558, *p* < 0.001) (Figure 5C). Immunohistochemical assays indicated that SOCS6 expression was lower in GC tissues that exhibited high miR-653-5p expression, as compared with those that had lower miR-653-5p expression (Supplementary Figure 3A). Evaluation of SOCS6 expression in xenografted subcutaneous tumors transfected with vectors containing a miR-653-5p inhibitor, a miR-653-5p mimic, or a negative control vector showed that the expression of SOCS6 was negatively regulated by miR-653-5p (Supplementary Figures 3B,C). To further characterize the interactions between SOCS6 and miR-653-5p in GC, we conducted dual-luciferase reporter assays. Luciferase reporters containing mutated SOCS6 sequences with altered binding sites for miR-653-5p were engineered and then transduced into BGC823 cells with a miR-653-5p mimic or a control (Figure 5D). Inhibition of wt-SOCS6-driven luciferase activity by a miR-653-5p mimic was abrogated by the mutated SOCS6 sequence. Furthermore, increased expression of miR-653-5p resulted in significantly decreased SOCS6 RNA and protein expression levels (Figures 5E,F). These results demonstrated that miR-653-5p could bind and negatively regulate the expression of SOCS6.

### SOCS6 Attenuated miR-653-5p-Mediated Promotion of GC Cell Growth

**FIGURE 6:**
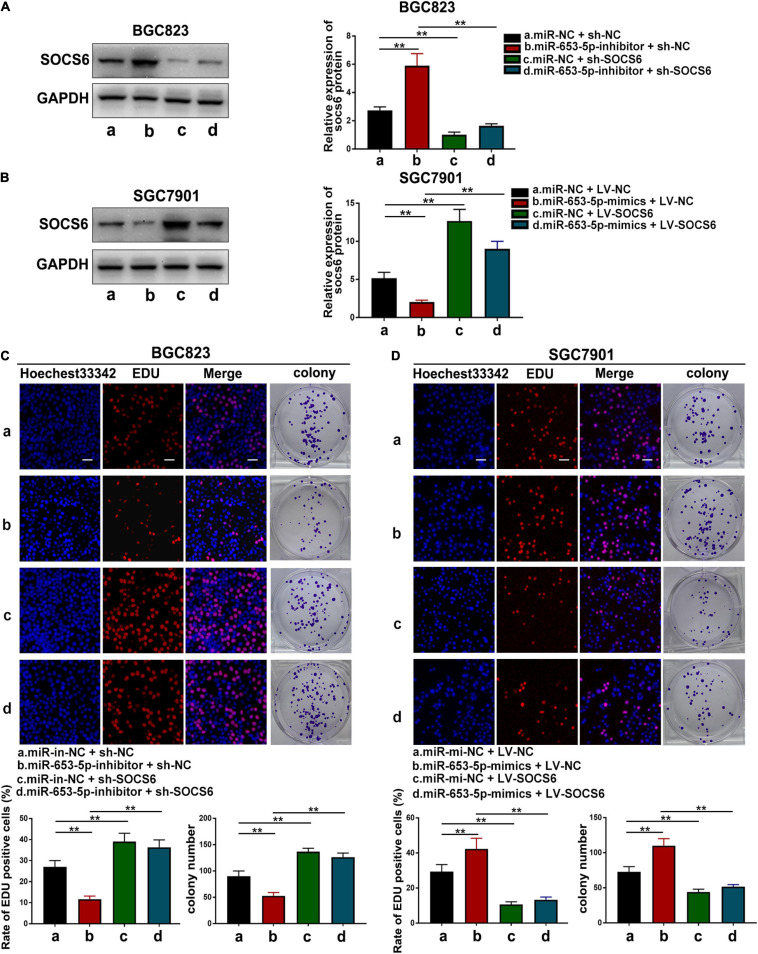
SOCS6 could rescue the effects of miR-653-5p on GC cell proliferation. **(A)** Inhibition of miR-653-5p rescued the downregulated expression of SOCS6. **(B)** Upregulation of miR-653-5p attenuated overexpression of SOCS6. **(C,D)** MiR-653-5p required SOCS6 to promote BGC823 **(C)** and SGC7901 **(D)** cell proliferation, as determined using EdU incorporation and colony formation assays; scale bar = 100 μm. Error bars show the mean ± SD. *, *p* < 0.05; **, *p* < 0.01; ***, *p* < 0.001.

**FIGURE 7:**
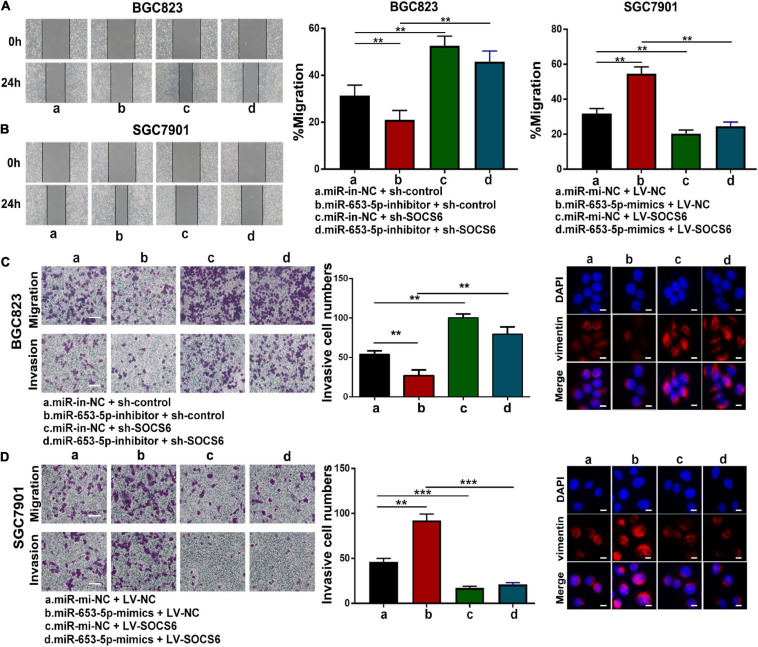
MiR-653-5p facilitated GC cell migration and invasion through targeting of SOCS6. **(A,B)** MiR-653-5p rescued the effects of SOCS6 on cell migration as indicated by the wound healing assay. **(C,D)** Metastatic ability of treated BGC823 cells **(C)** and SGC7901 cells **(D)** was measured using transwell **(left panel)** and immunofluorescence **(right panel)** assays, as indicated. Error bars show the mean ± SD. *, *p* < 0.05; **, *p* < 0.01.

To further investigate the role of miR-653-5p in GC, SOCS6 was silenced or overexpressed using a lentivirus vector (Figure 5G). As illustrated in Figure 6A, co-transfection with a miR-653-5p inhibitor rescued the expression of SOCS6 in BGC823 cells transfected with shRNA targeted to SOCS6. In contrast, overexpression of miR-653-5p inhibited the overexpression of SOCS6 in SGC7901 cells (Figure 6B). Results from EdU incorporation and colony formation assays, in which miR-653-5p-mediated stimulation of GC proliferation was inhibited by SOCS6, further supported these results (Figures 6C,D). Furthermore, wound healing assay showed that SOCS6 attenuated miR-653-5p-mediated promotion of GC cell migration (Figures 7A,B). Transwell and immunofluorescence assays indicated that miR-653-5p-driven cell invasion and migration were mitigated through the upregulation of SOCS6 (Figures 7C,D). These results indicated that the miR-653-5p-SOCS6 pathway may play a role in GC tumorigenesis and metastasis.

### MiR-653-5p Targeted the SOCS6/STAT3 Pathway *via* Downregulation of SOCS6

**FIGURE 8:**
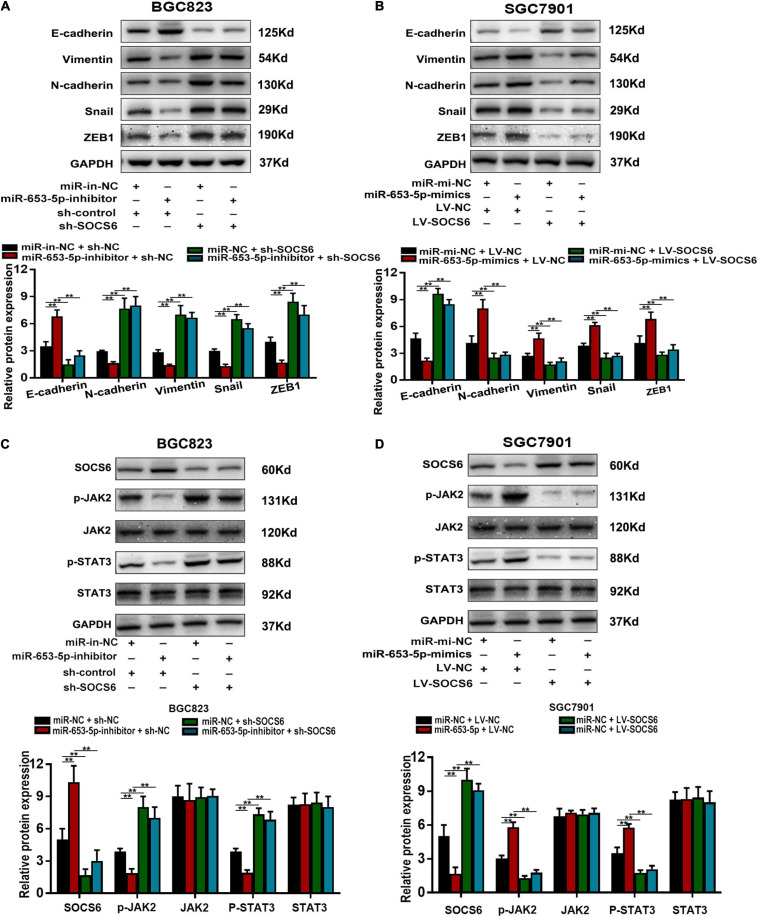
MiR-653-5p mediated GC progression by targeting the SOCS6-STAT3 pathway. **(A,B)** The expression of EMT-related proteins. **(C,D)** MiR-653-5p reversed SOCS6-mediated dephosphorylation of JAK2 and STAT3. Error bars show the mean ± SD. *, *p* < 0.05; **, *p* < 0.01; ***, *p* < 0.001.

The SOCS family contains key negative regulators of cytokine signaling, and the critical role of the JAK/STAT signaling cascade in cytokine function has been well-characterized (Krebs and Hilton, 2001). However, the role of SOCS6 in the regulation of JAK/STAT signaling is unclear. We co-transfected a miR-653-5p inhibitor and SOCS6-targeting shRNA into GC cells and determined the protein levels of EMT-related genes. The results showed that decreased expression levels of vimentin, N-cadherin, snail, and ZEB1 in response to a miR-653-5p inhibitor were significantly rescued by depletion of SOCS6 (Figure 8A). Furthermore, overexpression of SOCS6 inhibited miR-653-5p-mediated changes in expression levels of EMT-related genes (Figure 8B). To characterize potential interactions among miR-653-5p, SOCS6, and Janus kinase 2/signal transducer and activator of transcription 3 (JAK2/STAT3), the phosphorylation levels of JAK2 and STAT3 were evaluated in treated GC cells. As shown in Figures 8C,D, miR-653-5p increased phosphorylation of JAK2 and STAT3 but did not alter JAK2 and STAT3 protein levels, through downregulation of the expression of SOCS6. These results indicated that SOCS6 may be involved in the JAK2/STAT3 signaling pathway through miR-653-5p-mediated inhibition of JAK2 phosphorylation.

## Discussion

Studies over the previous decade have highlighted the importance of miRNAs as diagnostic biomarkers (i.e., miR-106b and miR-21) (Shiotani et al., 2013), prognostic indicators (i.e., miR-203) (Imaoka et al., 2016), and therapeutic targets (i.e., miR-375) (Xu et al., 2011) in GC. These miRNAs have been shown to be involved in GC proliferation, invasion, apoptosis, and chemoresistance. Integrated analysis of several classical online databases identified miR-653-5p as significantly upregulated in GC tissues and serum of patients with GC. Furthermore, miR-653-5p was strongly associated with GC prognosis. miRNA-653-5p, a typical multifunctional miRNA located on chromosome 7, has been reported to promote prostate cancer cell proliferation and invasion (Fu et al., 2019) and to restrict neuroblastoma progression (Chi et al., 2019). In GC, miR-653-5p has been shown to target circHIPK3 under chronic hypoxic conditions and attenuate the metastatic ability of hypoxia-resistant GC cell lines (Jin et al., 2020). These results were contradictory to the findings in this study. Differential expression of miR-653-5p in various cancers or in distinct microenvironments might occur due to the diverse features of cancers. Furthermore, previous studies only discussed the function and expression of miR-653-5p under the regulation of long non-coding RNA or circular RNA. Moreover, Luo et al. (2019) reported that an 11-miRNA expression signature including miR-653 was as an independent predictor of poor prognosis, and was an excellent predictor of overall survival. Importantly, this study strongly suggested that miR-653-5p promoted GC cell proliferation and metastasis both *in vitro* and *in vivo*, which indicates miR-653-5p may play an important role in GC progression.

The underlying mechanisms responsible for changes in the expression of miRNAs in diseases include genomic events (i.e., mutations and deletion) and downregulation of enzymes that regulate miRNA synthesis (Bertoli et al., 2015). Recently, other non-coding RNAs, such as long non-coding RNA and circular RNAs, were identified and were shown to modulate the expression levels of miRNAs by acting as miRNA sponges (Ebert and Sharp, 2010). Furthermore, exosome-mediated delivery of functional miRNAs is believed to be involved in disease progression and has been shown to stimulate angiogenesis and facilitate metastasis in cancer (Sun et al., 2018). A deeper understanding of the mechanisms responsible for changes in expression of miR-653-5p may help to identify novel diagnostic and therapeutic targets for GC.

Although a single miRNA can regulate a set of genes responsible for a particular malignant phenotype, we selected SOCS6 as the downstream target of miR-653-5p. The suppressor of cytokine signaling proteins family, of which SOCS6 is a member, is involved in a classical negative feedback circuit for cytokine signaling and has been implicated in development of various diseases, including cancer (Linossi et al., 2013; Jiang et al., 2017). In this study, loss of SOCS6 inhibited GC cell proliferation and metastasis. Moreover, SOCS6-mediated inactivation of STAT3 was reversed by miR-653-5p. Cytokine signaling induces activation of JAK/STAT pathways, which results in the upregulation of gene transcription (Rawlings et al., 2004). However, SOCS6 was initially believed to be less involved in JAK/STAT signaling than SOCS1 or SOCS3. Recent evidence suggests that SOCS6 can inactivate the JAK2/STAT3 pathway in tumor cells (Gong et al., 2017). In GC, we hypothesize that SOCS6 may affect phosphorylation of JAK2, but not degrade JAK2 protein, to inhibit JAK2/STAT3 signaling. Future experiments will focus on evaluation of the role of SOCS6 in the JAK2/STAT3 signaling pathway. Furthermore, a high rate of methylation was detected on several SOCS genes, such as SOCS1 in hepatocellular carcinoma (Okochi et al., 2003), SOCS3 in head and neck squamous cell carcinoma (Weber et al., 2005), and SOCS6 in GC (Lai et al., 2010). Thus, downregulation of SOCS6 may play a vital role in GC carcinogenesis.

In conclusion, we identified miR-653-5p as a prognostic miRNA that promoted GC cell proliferation and metastasis. Mechanistically, SOCS6 was a direct target of miR-653-5p, and SOC6-mediated inactivation of JAK2/STAT3 was rescued by miR-653-5p. Our results showed that miR-653-5p promoted the development and progression of GC through inhibition of the SOCS6-STAT3 pathway.

## Data Availability Statement

The original contributions presented in the study are included in the article/Supplementary Material, further inquiries can be directed to the corresponding authors.

## Ethics Statement

The studies involving human participants were reviewed and approved by the Ethics Committee of the First Affiliated Hospital of Nanjing Medical University. The patients/participants provided their written informed consent to participate in this study. The animal study was reviewed and approved by Committee on the Ethics of Animal Experiments of the Nanjing Medical University.

## Author Contributions

ZL: study concept and design, acquisition of data, analysis, and interpretation of data, statistical analysis, and drafting of the manuscript. HF: acquisition of data, analysis, interpretation of data, and statistical analysis. WC, XM, and PN: acquisition of data. JX: critical revision of the manuscript. ZX: study supervision. LY: obtaining funding, critical revision of the manuscript for important intellectual content, and study supervision. All authors reviewed and approved the manuscript.

## Conflict of Interest

The authors declare that the research was conducted in the absence of any commercial or financial relationships that could be construed as a potential conflict of interest.

## Footnotes

1 http://www.oncomir.org

2 https://portal.gdc.cancer.gov

3 https://www.ncbi.nlm.nih.gov/gds

4 http://kmplot.com/analysis

5 http://www.targetscan.org/

6 http://starbase.sysu.edu.cn

7 http://zmf.umm.uni-heidelberg.de/apps/zmf/mirwalk2/

8 https://www.isical.ac.in/~bioinfo_miu/targetminer20.htm
