# Supplementary material for: MicroRNA-653-5p Promotes Gastric Cancer Proliferation and Metastasis by Targeting the SOCS6-STAT3 Pathway
Source: Front Mol Biosci. 2021 Apr 15;8:655580. doi: 10.3389/fmolb.2021.655580 (PMC8082248; doi:10.3389/fmolb.2021.655580)
Supplement: Supplementary file 1 [file Table_1.DOCX]

**Supplementary Figure 1. MiR-653-5p was overexpressed in gastric cancer tissues and correlated with poor prognosis of gastric cancer patient.**

**
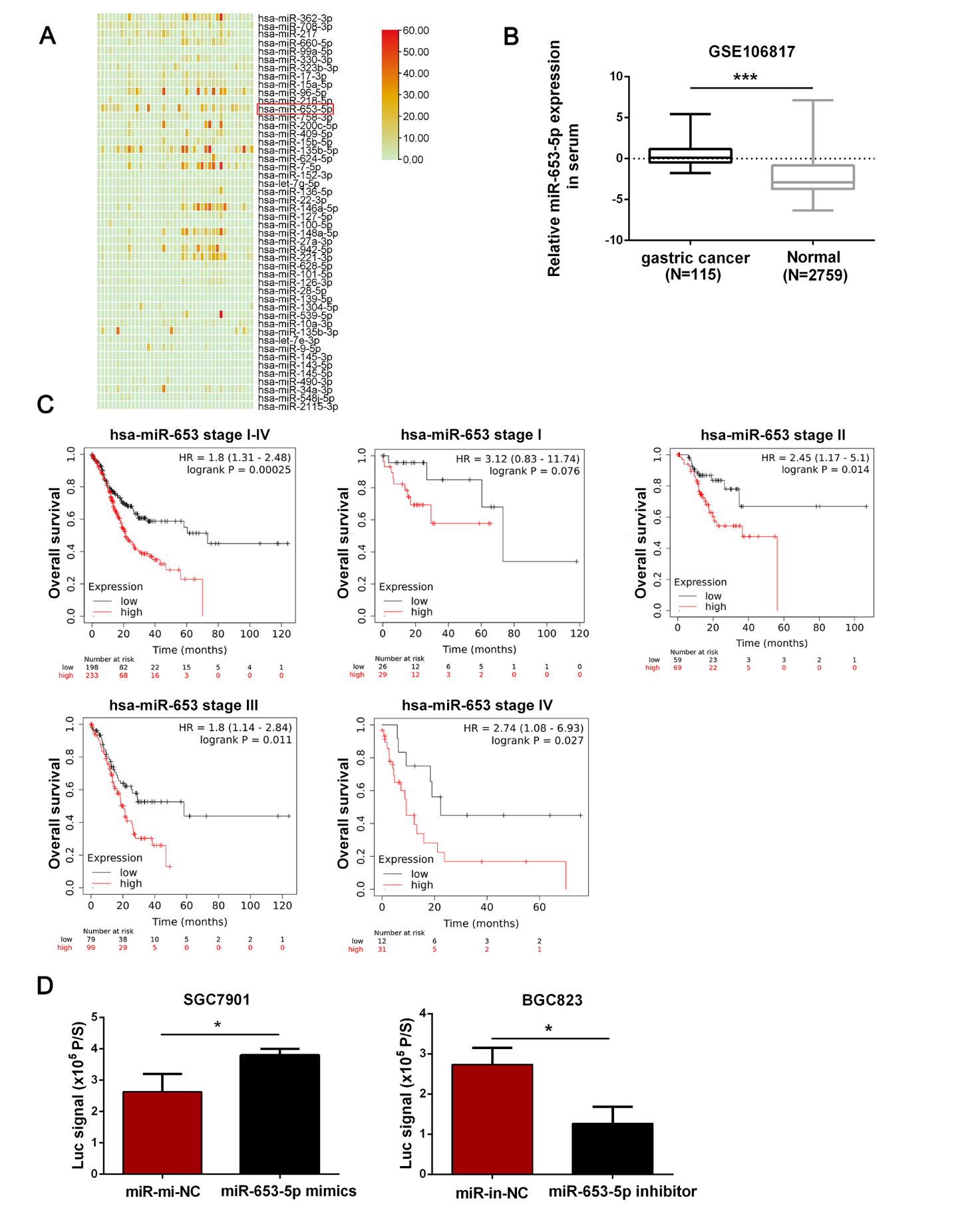
**

**Figure S1. MiR-653-5p was overexpressed in gastric cancer tissues and correlated with poor prognosis of gastric cancer.** **A**. Heatmap presentation of the expression of 48 prognosis-related miRNAs in 41 paired gastric cancer tissues from TCGA database. **B.** The expression of miR-653-5p in gastric cancer serum and normal serum. **C.** MiR-653 predicts poor overall survival in GC patients with stage II, III, IV, I-IV from KM plotter. **D.** Nude mice (n=4) were injected via their tail vein with indicated cells (0.5x10^6^), and changes in bioluminescence signals from mice were measured using the IVIS200 Imaging System. Error bars, mean ± SD.*, P < 0.05; **, P < 0.01; ***, P < 0.001.

**Supplementary Figure 2. The associations with gastric cancer overall survival of SOCS6.**


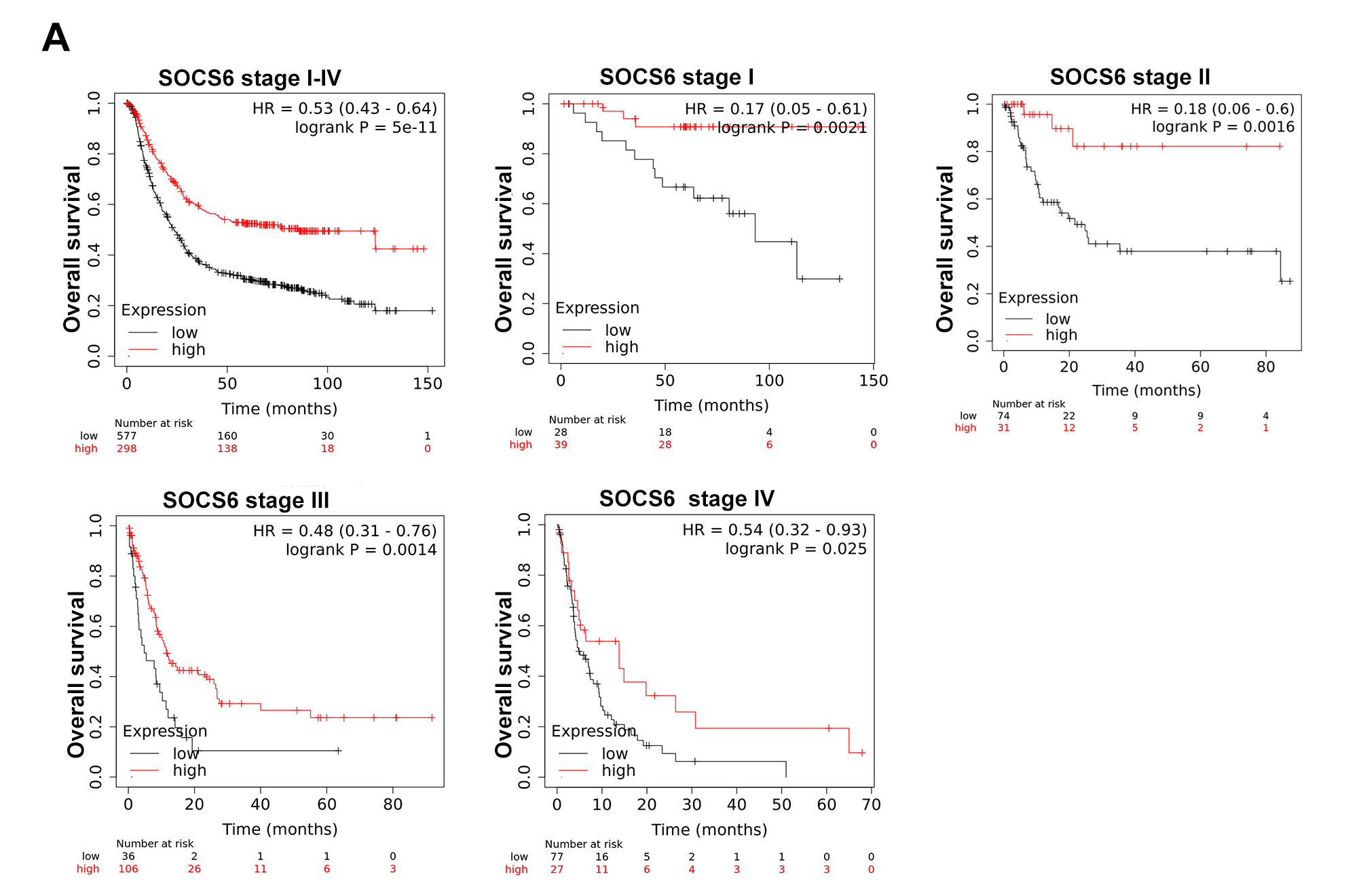


**Figure S2. The associations with gastric cancer overall survival of SOCS6.** **A**. SOCS6 is associated with better overall survival in GC patients with stage I, II, III, IV, I-IV from KM plotter.

**Supplementary Figure 3. The expression of SOCS6 was negatively regulated by miR-653-5p.**


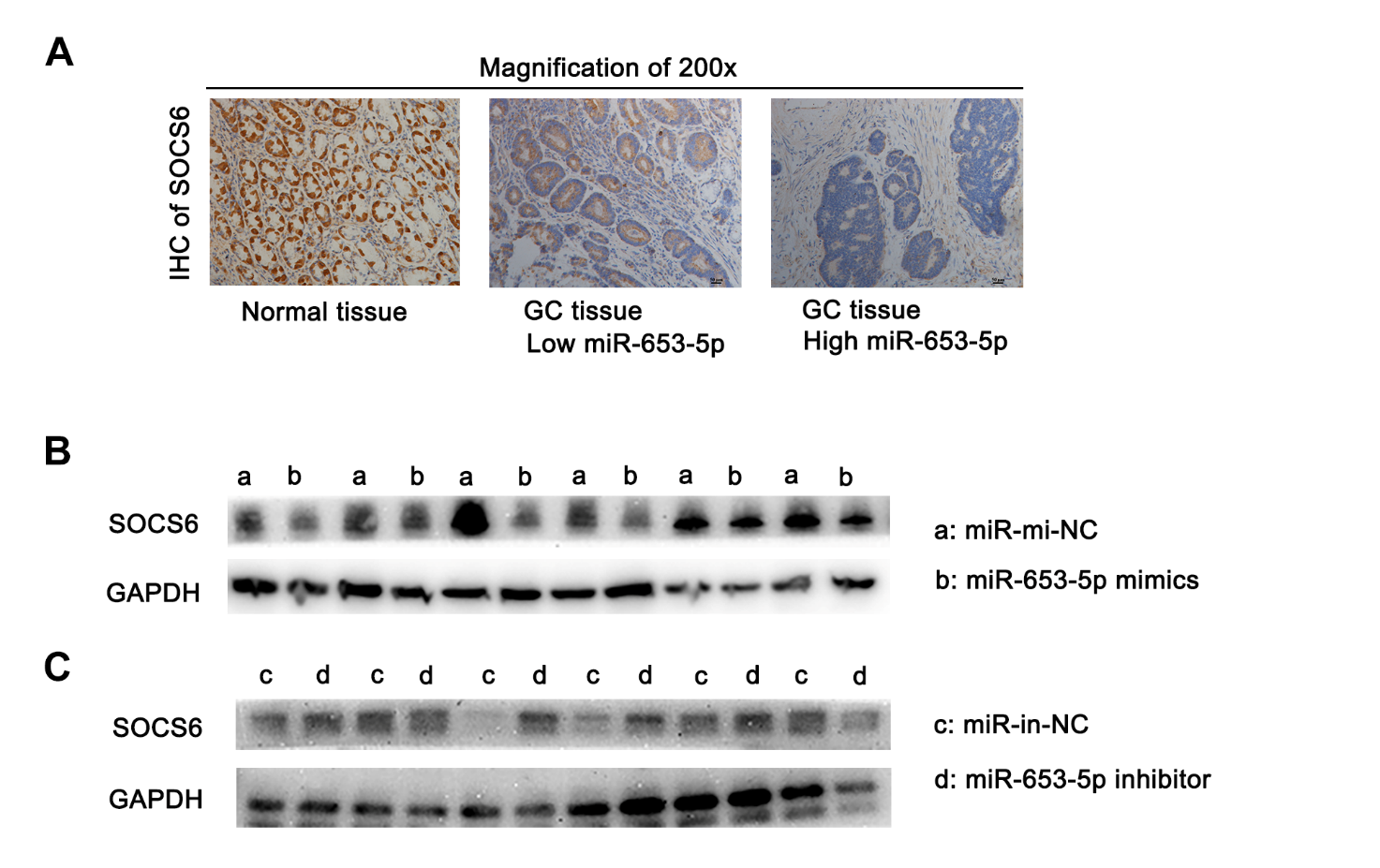


**Supplementary Figure 3. The expression of SOCS6 was negatively regulated by miR-653-5p. A.** Representative images of the expression and distribution of SOCS6 protein in gastric cancer tissue and corresponding normal tissue by IHC. **B-C**. the expression of SOCS6 in xenografted subcutaneous tumors that transfected with vector contains miR-653-5p mimics (**B**), miR-653-5p inhibitor (**C**), or negative control.

**Supplementary Table 1. Primers used for quantitative RT-PCR.**

| RNA | 5' to 3' |
| --- | --- |
| miR-653-5p | Forward TTGAAACAATCTCTACTGAACCAA |
|  | Universal reverse GCGAGCACAGAATTAATACGAC |
| U6 | Forward CTCGCTTCGGCAGCACA |
|  | Universal reverse GCGAGCACAGAATTAATACGAC |
| SOCS6 | Forward AAGAATTCATCCCTTGGATTAGGTAAC |
|  | Reverse CAGACTGGAGGTCGTGGAA |
| GAPDH | Forward TGCACCACCAACTGCTTAGC |
|  | Reverse GGCATGGACTGTGGTCATGAG |

**Supplementary Table 2. Antibodies used for Western blot.**

| antibody | company/provider (Dilution ratio) |
| --- | --- |
| GAPDH | Proteintech, Wuhan, China (1:50000) |
| anti E-Cadherin | Proteintech, Wuhan, China (1:2000) |
| anti-Vimentin | Proteintech, Wuhan, China (1:2000) |
| anti-c-Myc | CST, Danvers, MA, USA (1:2000) |
| anti-N-Cadherin | Proteintech, Wuhan, China (1:2000) |
| anti-Snail | Proteintech, Wuhan, China (1:2000) |
| anti-ZEB1 | Proteintech, Wuhan, China (1:2000) |
| anti-SOCS6 | Abcam, CA, MA, USA (1:2000) |
| anti-STATA3 | Abcam, CA, MA, USA (1:2000) |
| anti-[STAT3 (phosphoY705)](https://www.abcam.cn/stat3-phospho-y705-antibody-ep2147y-ab76315.html) | Abcam, CA, MA, USA (1:2000) |

**Supplementary Table 3. Associations between miR-653-5p expression and clinicalpathological features of gastric cancer.**

| characteristics | Group | Cases (n=58) | | p-value |
| --- | --- | --- | --- | --- |
|  |  | Low expression | High expression |  |
| Age | <60 | 7 | 19 | 0.291 |
|  | ≥60 | 5 | 27 |  |
| Gender | Male | 7 | 33 | 0.371 |
|  | Female | 5 | 13 |  |
| T stage | Tis-T1 | 4 | 9 | 0.308 |
|  | T2-T4 | 8 | 37 |  |
| Lymph node metastasis | Absent(N0) | 6 | 5 | **0.002** |
|  | Present(N1-N3) | 6 | 41 |  |
| TNM stage | I-II | 5 | 7 | **0.044** |
|  | III-IV | 7 | 39 |  |
| Location | Cardia | 3 | 17 | 0.438 |
|  | Non-cardia | 9 | 29 |  |

Clinicopathological results were compared using Pearson χ2 tests.

The significant results are in bold.
