# Supplementary figures and images for: MicroRNA-653-5p Promotes Gastric Cancer Proliferation and Metastasis by Targeting the SOCS6-STAT3 Pathway
Source: Front Mol Biosci. 2021 Apr 15;8:655580. doi: 10.3389/fmolb.2021.655580 (PMC8082248; doi:10.3389/fmolb.2021.655580)

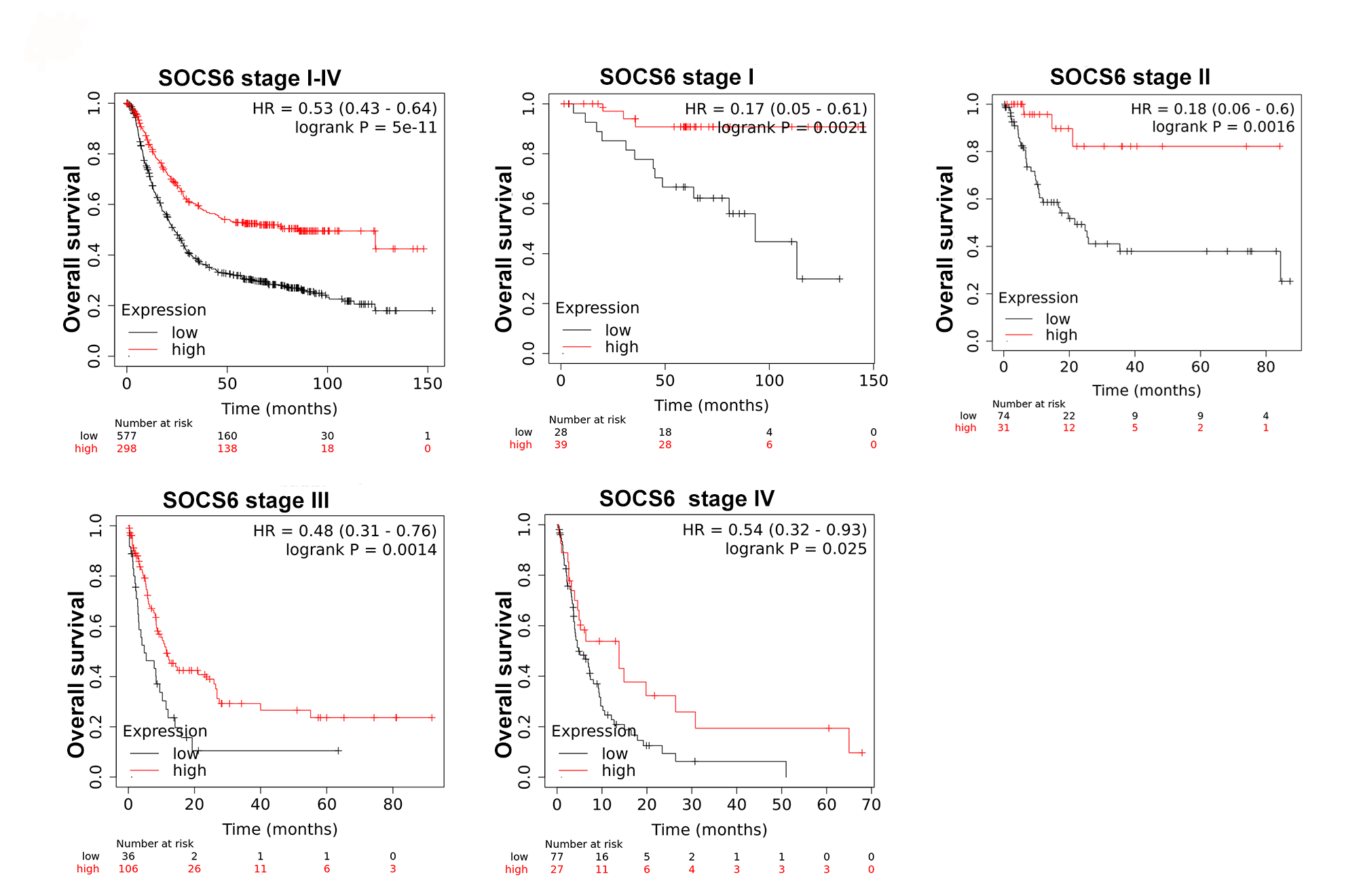

Supplement: Supplementary file 2 [file Image_1.tif]
